# Rare case of beet juice mimicking gastrointestinal bleeding: diagnostic implication

**DOI:** 10.1055/a-2268-6016

**Published:** 2024-03-08

**Authors:** Byung Chul Jin, Seung Young Seo, Sang-Wook Kim

**Affiliations:** 1 90158 Department of Internal Medicine, Jeonbuk National University Medical School, Jeonju, Korea (the Republic of)

Lower gastrointestinal hemorrhage has demonstrated an increasing trend in morbidity and mortality, classifying it as a potential cause of life-threatening events 1 . In cases of acute intestinal bleeding in the large intestine, a colonoscopy should be performed when the patient is stable to identify the cause. This case highlights the importance of comprehensive patient interviews before diagnostic procedures.

A 68-year-old man with a history of chronic constipation was taking mosapride and magnesium hydroxide. He had no other significant medical history and underwent a screening colonoscopy. The patient was hospitalized for diverticulitis 10 years ago and underwent a hemorrhoidectomy 6 years ago. At the last colonoscopy 2 years ago, several diverticula were observed in the ascending colon, and one hyperplastic polyp was also removed.

For bowel preparation, the patient used a split-dosing regimen of 2-L low-dose polyethylene glycol, specifically Cleanblueall powder, and reported no stool abnormalities during preparation. On the morning of the test, the patient called to ask if it was permissible to consume electrolyte drinks and received positive advice from the nurse. Lower gastrointestinal bleeding was suspected; a colonoscopy was performed that morning 2 .

Multiple fresh and red diverticula were visualized in the ascending colon, despite being mixed with normal stool ( Fig. 1 , Fig. 2 , Fig. 3 ). Yellow fluid was observed in the descending colon and sigmoid colon ( Fig. 4 , Fig. 5 ); no bleeding areas were identified ( Video 1 ). Thereafter, blood pressure stabilized at 120/70 mmHg, pulse rate stabilized at 70 bpm, and the patient appeared comfortable. The patient later mentioned consuming mixed beets before the morning colonoscopy, mistaking them for a commercial electrolyte drink, to rehydrate and replenish electrolytes.

**Fig. 1:**
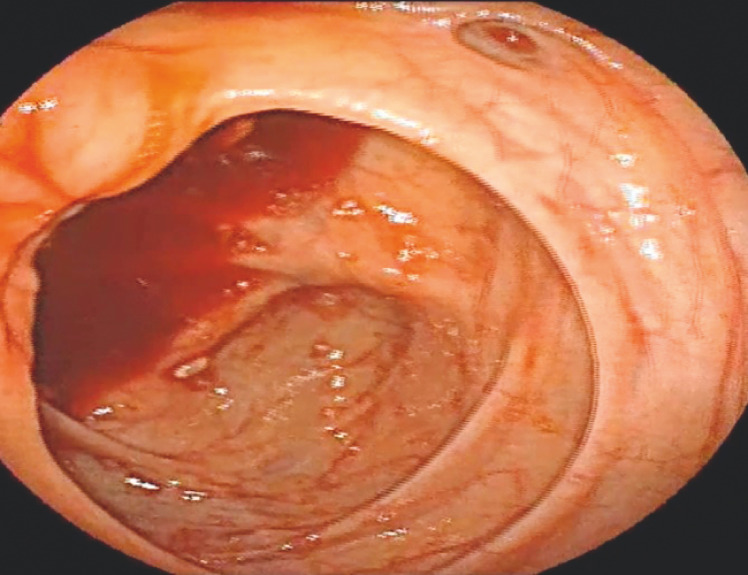
Colonoscope at the cecum.

**Fig. 2:**
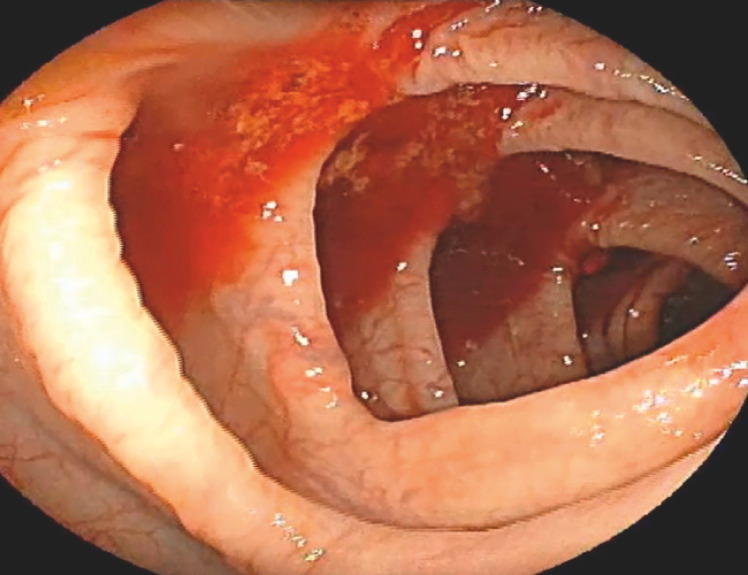
Colonoscope at the ascending colon.

**Fig. 3:**
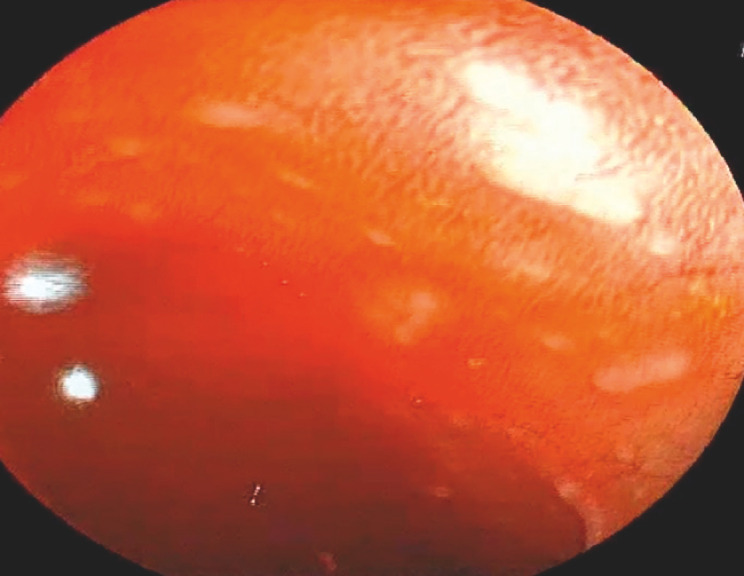
Colonoscope at the ileocecal valve.

**Fig. 4:**
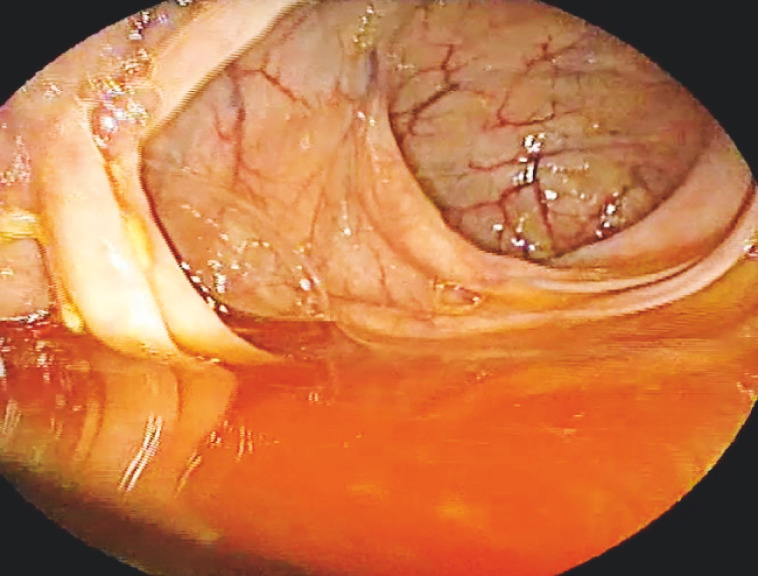
Colonoscope at the descending colon.

**Fig. 5:**
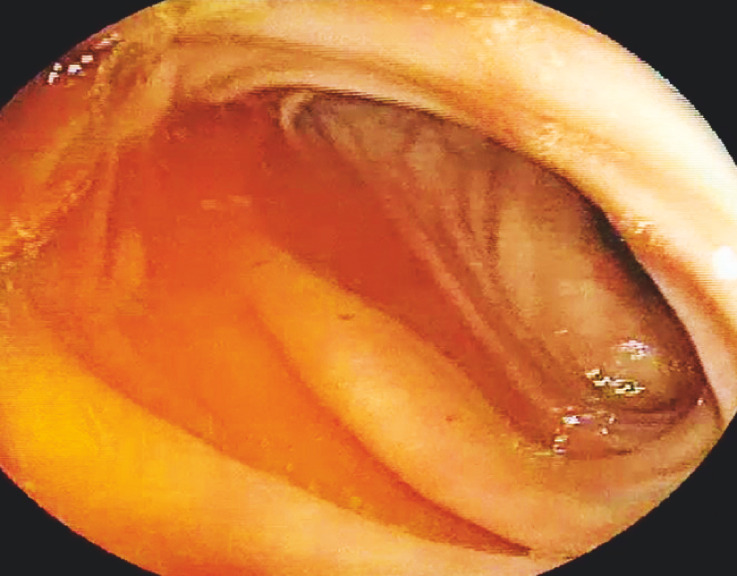
Colonoscope at the sigmoid colon.

A rare case of beet juice mimicking gastrointestinal bleeding.Video 1

A 1-day observation revealed red fluid in the stool without any signs of active bleeding. This case highlights the possibility of misinterpreting beet consumption as gastrointestinal bleeding due to their similar color to blood.

Endoscopy_UCTN_Code_TTT_1AQ_2AZ

## Notes

Endoscopy E-Videos https://eref.thieme.de/e-videos
*E-Videos* is an open access online section of the journal *Endoscopy* , reporting on interesting cases and new techniques in gastroenterological endoscopy. All papers include a high-quality video and are published with a Creative Commons CC-BY license. Endoscopy E-Videos qualify for HINARI discounts and waivers and eligibility is automatically checked during the submission process. We grant 100% waivers to articles whose corresponding authors are based in Group A countries and 50% waivers to those who are based in Group B countries as classified by Research4Life (see: https://www.research4life.org/access/eligibility/ ). This section has its own submission website at https://mc.manuscriptcentral.com/e-videos .
